# What is the analgesic range of acupuncture stimulus for treating acute pain?

**DOI:** 10.3389/fnhum.2023.1281832

**Published:** 2023-11-02

**Authors:** Kwang-Ho Choi, Seong Jin Cho, Minji Kim, O Sang Kwon, Suk-Yun Kang, Su Yeon Seo, Se Kyun Bang, Yeonhee Ryu

**Affiliations:** ^1^KM Science Research Division, Korea Institute of Oriental Medicine, Daejeon, Republic of Korea; ^2^College of Korean Medicine, Wonkwang University, Iksan, Jeonbuk, Republic of Korea

**Keywords:** acupuncture stimulation, acute pain, somatosensory evoked potentials, EEG, verbal rating scale, analgesic effect, sensory threshold

## Abstract

**Introduction:**

Since the analgesic effect of acupuncture stimulation is derived from different mechanisms depending on the type of pain, it is important to know which acupuncture points to stimulate. In this study, to confirm the effect of acupuncture stimulation on acute pain from a neurological point of view, somatosensory evoked potential and sensory threshold changes were evaluated to identify the nerve range that is affected by acupuncture stimulation on LI4 (Hapgok acupuncture point, of the radial nerve) during acute pain.

**Methods:**

The subjects were 40 healthy men and women aged 19–35 years. The study was designed as a randomly controlled, crossover trial with acupuncture stimulation at LI4 as the intervention. The washout period for acupuncture stimulation was 2 weeks, and the subjects were divided into two groups, i.e., an acupuncture stimulation group and a nonstimulation group, with 10 men and 10 women in each group. Somatosensory evoked potential measurement was carried out for 5 min by alternately applying 2 HZ-pulse electrical stimulation to the thumb and the little finger of the hand acupunctured with a 64-channel electroencephalogram. The verbal rating scale was used before and after each acupuncture stimulation session.

**Result and discussion:**

The results of the study confirmed that the somatosensory evoked potential amplitude value of the thumb was significantly decreased and that the intensity of sensory stimulation corresponding to a verbal rating scale score of 6 was significantly increased only in the thumb after acupuncture stimulation. Therefore, the results show that acupuncture treatment for acute pain is more effective when direct acupuncture stimulation is applied to the painful area.

## 1. Introduction

Because acupuncture is effective in alleviating a variety of acute pains and chronic pains (Lin et al., 2022), it has been widely used to alleviate pain, not only in Asian countries, but also, worldwide (Zhao, 2008). However, there has long been controversy over whether the analgesic effect of acupuncture is nothing more than a placebo effect induced by the individual patient’s psychological factors (Enck et al., 2010; Zheng et al., 2014). Therefore, since the analgesic effect of acupuncture is related to the feeling and sensation of pain caused by the nerve reaction from the skin to the brain, researchers have been trying to investigate the central and peripheral nerve mechanisms among various physiological mechanisms.

Acupuncture analgesia is a manifestation of an integrative process of afferent impulses in the pain area and of acupuncture point impulses with different levels in the CNS (Takeshige et al., 1993), and the segmental mechanism of the spinal cord between the pain area and the acupuncture point supports the functional specificity of acupuncture points (Bing et al., 1991). As reported thus far, in manual acupuncture (MA), all types of afferent fibers (Aβ, Aδ, and C) are activated and transmit signals (Bing et al., 1990; Okada et al., 1996; Zhu et al., 2004), whereas in electroacupuncture, the analgesic effect can be induced if parts of the Aβ-type afferents (group II) and Aδ-type afferents (group III) are stimulated by a sufficiently strong current (Levine et al., 1976; Chung et al., 1984; Leung et al., 2005). It is known that the pathway of the spinal cord for acupuncture stimulation (AS) at the acupuncture point is mainly via the ventrolateral funiculus to the brain and via some areas of the brain, such as the rostral ventromedial medulla (mainly the nucleus raphe magnus, NRM), the locus coeruleus, the periaqueductal gray, the preoptic area, and the amygdala, and that many complicated networks of the brain’s structure, including various nuclei, are involved in the acupuncture analgesia process (Zhao, 2008). At present, it has been experimentally proven that the habenula area of the brain interrupts the analgesic effect of acupuncture (Wang et al., 1987; Liu and Wang, 1988). Additionally, it is known that glutamate (NMDA and AMPA/KA receptor) (Zhang et al., 2002; Huang et al., 2004), opioid peptides (Cao, 2002; Han, 2004), cholecystokinin octapeptide (Huang et al., 2007), 5-hydroxytryptamine (Qu and Zhou, 2007) and noradrenaline (Murotani et al., 2010), among neurotransmitters and neuromodulators, are involved in acupuncture analgesia.

The analgesic effect of AS has different mechanisms depending on the type of pain and thus depends on both the sites of the acupuncture points and the mode of stimulation. Several sessions are required to obtain accumulative effects in treating chronic pain, while it is effective to stimulate the acupuncture point of the relevant meridian because the source of acute pain is only maintained for a short period of time (Chang, 2013). However, the grounds for stimulating the relevant acupuncture points in the acute pain are insufficient.

Thus, this study was performed to investigate the effects of AS at LI4 of the radial nerve on relevant and irrelevant nerves to establish firm grounds for stimulating the acupuncture points related to acute pain by comparing somatosensory evoked potential (SEP) and sensory threshold changes and identifying the nerve range affected by AS. In addition, as disputes concerning the role of the autonomic nervous system in explaining the analgesic effect of acupuncture have continued (Anderson et al., 2012; Chung et al., 2014), the role of the autonomic nervous system in acupuncture analgesia was also investigated by comparing heart rate variability (HRV) changes induced by AS.

## 2. Materials and methods

### 2.1. Study design

A randomized, controlled, crossover clinical trial was designed for the intervention of AS at LI4. The total study period was up to 4 weeks, and the washout period was at least 2 weeks. The subjects were divided into two groups of 20 (each containing 10 males and 10 females), and the order of intervention was randomly assigned. The study was performed at the Daejeon Korean Medicine Hospital affiliated with Daejeon University, Daejeon, Korea, with IRB approval (No. djomc-129-1) according to the Declaration of Helsinki and registered at the Clinical Research Information service under the registration number KCT0001865.^1^

### 2.2. Participants

The subjects of the study were 40 healthy individuals, 20 men and 20 women aged 19 ~ 35 years. They were recruited at the Daejeon Korean Medicine Hospital affiliated with Daejeon University between September 11, 2015, and March 10, 2016. All the participants were instructed and agreed to sign the consent form. The subject information is presented in Table 1, and the inclusion and exclusion criteria are as described below.

**Table 1:** Subject information.

	Age	Height	Weight
Male	22.79 ± 2.04	172.07 ± 5.35	70.94 ± 10.04
Female	22.10 ± 2.51	161.28 ± 5.43	52.65 ± 6.57
Total	22.44 ± 2.28	166.54 ± 7.58	61.08 ± 12.04

#### 2.2.1. Inclusion criteria for all participants

1. Individuals without any abnormal findings in their blood pressure and Photoplethysmography (PPG) test. The criteria for abnormal findings were as follows: blood pressure measured twice and averaged in a sitting position, at intervals of at least 2 min after resting for at least 5 min; systolic blood pressure of 160 mmHg and over, or diastolic blood pressure of 100 mmHg and over; arrhythmia in the PPG test. 2. Individuals who were clearly informed about the purpose and characteristics of the trial and who agreed to participate in it signed the consent form.

#### 2.2.2. Exclusion criteria for all participants

1. Individuals with autoimmune diseases such as asthma. 2. Individuals with skin allergies such as atopy. 3. Individuals with accompanying conditions that can affect the head, neck, and facial electromyogram (common cold, head injury, neck and jaw pain, toothache, or eye diseases). 4. Individuals who have undergone surgery within the last year or who have a medical history that includes serious health conditions. 5. Individuals with metal prostheses inserted in the body. 6. Individuals currently on medication (oriental medicine and Western medicine) or who plan to take medication during the clinical trial. 7. Individuals who are judged unable to maintain a sitting position for at least one hour. 8. Individuals who are unsuitable for low-frequency electric stimulation due to systemic skin disorders or an allergy to electrode paste. 9. Individuals who are judged unable to fill out the forms related to research performance. 10. Individuals with a medical history in psychiatry or with brain-related disorders. 11. Individuals who are judged unable to participate in the experiment easily due to their biorhythms (lack of sleep; drinking alcohol within 72 h). 12. Individuals who are judged by the investigators to be unsuitable to participate in the clinical trial.

### 2.3. Measurement

#### 2.3.1. Setting

All the subjects were prohibited from drinking alcohol, smoking cigarettes, and taking caffeine 24 h before the experiment and had to get enough sleep before participating in the experiment. Prior to the experiment, the subjects rested in the laboratory for 30 min without any stimulation under dim lighting to create the same preexperimental psychological condition for all the subjects.

#### 2.3.2. Procedure

**Figure 1:**
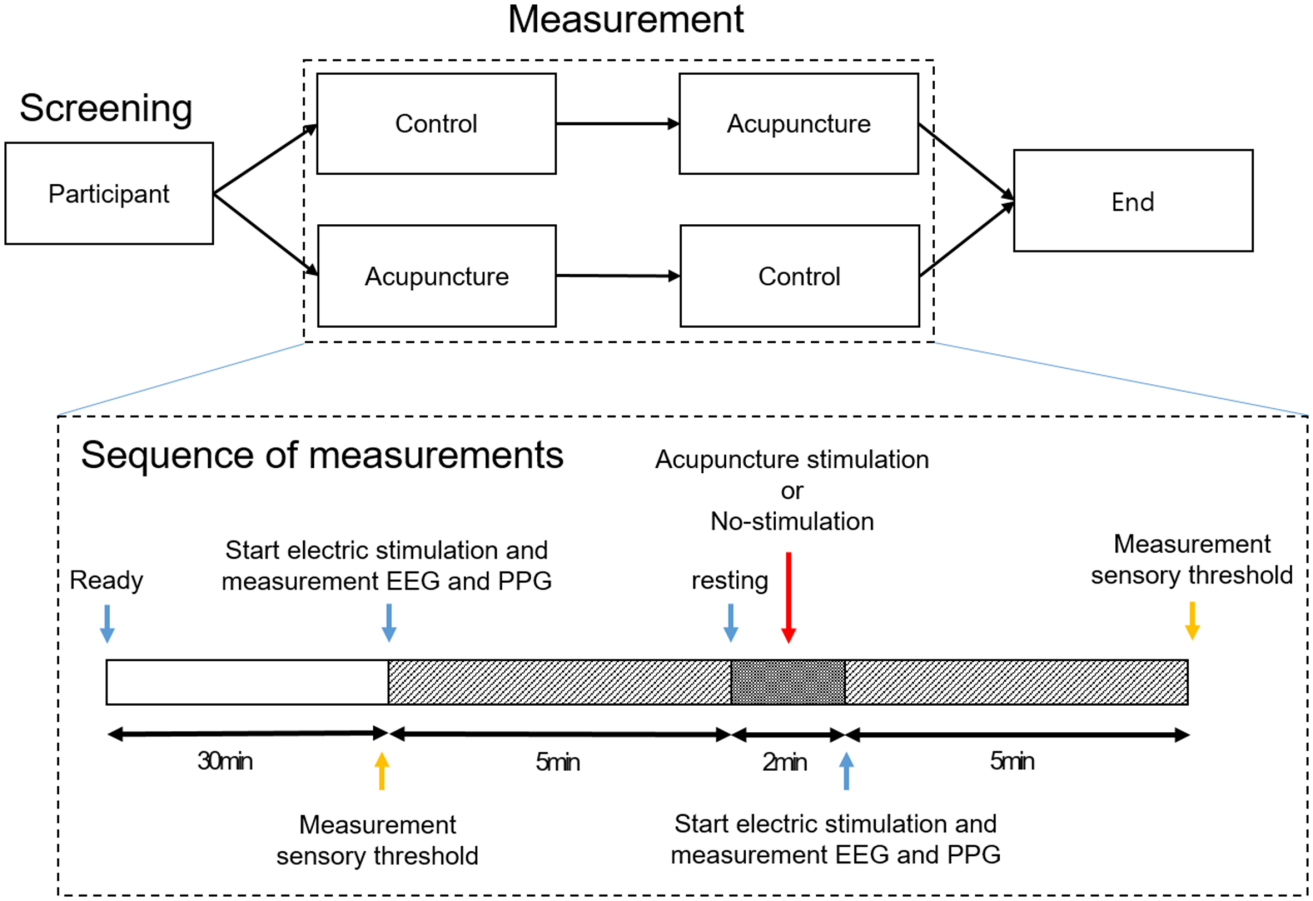
Experimental procedure.

While the subjects rested for 30 min, the setting for the SEP and PPG measurements was prepared. When the experiment started, SEP and PPG were measured for 5 min while the subjects closed their eyes. After the first measurement, the subjects opened their eyes and took a 2-min rest with either AS or no stimulation, and then SEP and PPG were measured for 5 min again with the eyes closed. Each subject underwent two experiments with AS and no stimulation, and the order of the experiment was randomly assigned using envelopes containing randomization information (See Figure 1). Envelopes for random assignment were created using a python program in advance by the researcher who analyzes the data.

#### 2.3.3. SEP measurement

SEPs are tests that are conducted to evaluate the potential evoked at the cervical spine and the sensory nerve center by applying bipolar transcutaneous electrical stimulation to the surface of a generally selected nerve and are the most widely used of all intraoperative neurophysiological monitoring tests (Cruccu et al., 2008). At this time, the waveform is obtained through the equalization of repeated stimulation, and nerve responses and/or nerve damage are evaluated by observing the shape or changes of the waveform. In this study, SEP measurement was performed to evaluate the threshold at which the sensory nervous system is changed or affected by AS.

EEG was measured using a Biosemi ActiveTwo system (Biosemi, Netherlands, https://www.biosemi.com/activetwo_full_specs.htm), with 64 preamplified Ag/AgCl electrodes mounted on the cap according to the 10–20 layout provided by the company. The amplifier of this system was DC-coupled, and electrodes were installed inside the plastic holder of the cap, which was filled with electrolyte gel. Data were digitalized at a sampling rate of 512 Hz.

**Figure 2:**
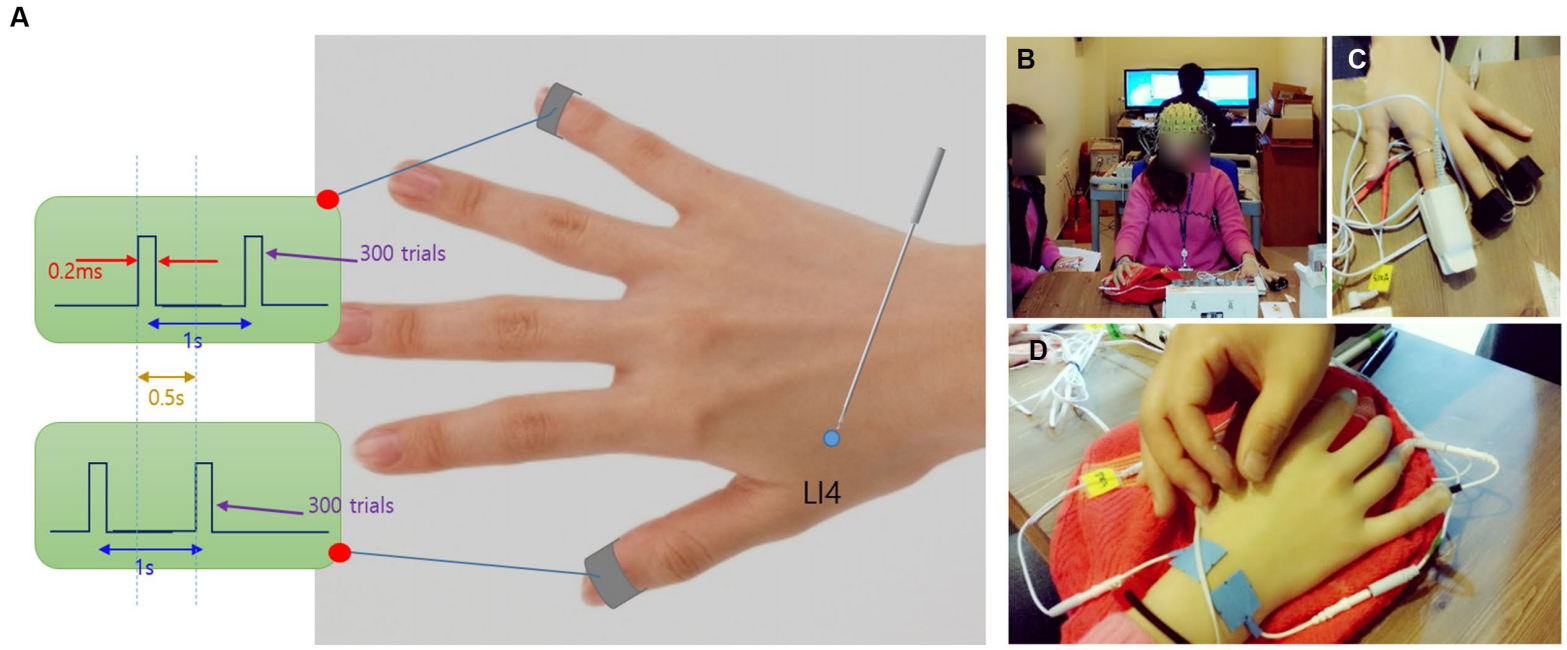
**(A)**. Bipolar transcutaneous electrical stimulation; **(B)**. EEG measurement; **(C)**. PPG measurement; **(D)**. AS.

Regarding electrical stimulation for the SEP measurement, an external electrode (Bio-Protech Inc., KOREA) of the kind generally used in low-frequency electrical stimulators was cut into 20 mm × 20 mm pieces and attached to the tip of the thumb and little finger of each subject’s right hand, and each opposite electrode was attached to the subject’s wrist (back of the hand side). The electrical stimulator was a PG-306 (Suzuki, Japan), and the electrical stimulation was of the pulse type and used a diode, which was set to stimulate the thumb and little finger, each at 1-s intervals and at 0.5 s alternately (See Figure 2). The pulse was a square wave with a duration of 0.2 ms, and each subject received electrical stimulation at an intensity of VAS 4 (i.e., the degree of intensity at which the subject starts to feel pain) on the thumb and little finger, each of which was checked before the experiment, and the same intensity was applied when the experiment started.

#### 2.3.4. PPG measurement

PPG was measured to indirectly identify the degree of sympathetic nerve activation by evaluating HRV before and after intervention. The PPG was measured using a complex polygraph system (PolyG-A, LAXTHA, KOREA) and measured at the same time that the EEG was measured by putting it on the index finger of the left hand.

#### 2.3.5. Measurement of changes in the sensory threshold

verbal rating scale (VRS) measurement was designed to evaluate changes in the sensory threshold of the thumb and little finger before and after intervention. Through the electric stimulation pad for SEP measurement attached to the thumb and little finger of each subject, the degree of stimulation inducing the same level of pain was evaluated. To evaluate pain-inducing stimulation, the degree of electrical stimulation that induced VRS 6 pain (pain of medium intensity) in each subject was recorded. The above sequence was performed before the first brainwave measurement after setting up the experiment and then after removing the needle immediately after the end of the experiment (See Figure 1).

### 2.4. Interventions

The subjects were divided into two groups, the AS group and nonstimulation (NS) group, and each subject underwent two experiments. AS and NS were performed over a period of at least 2 weeks for washout. AS was performed during a 2-min resting period after the first 5-min brainwave measurement, and a coated needle with a length of 40 mm and a thickness of 0.25 mm (acupuncture needle, Dongbang Acupuncture, Korea) was inserted into the LI4 of the right hand.

The location of LI4 (WHO STANDARD ACUPUNCTURE POINT LOCATION IN THE WESTERN PACIFIC REGION) was on the dorsum of the hand, radial to the midpoint of the second metacarpal bone. After inserting the needle, a doctor of oriental medicine flicked the needle’s handle with the middle finger to manipulate a total of 5 sets at a speed of 5 Hz, 3 times at 1 ~ 2-s intervals. After 2 min of rest, the brainwave was measured for 5 min, and the needle was removed. For the NS group, the experiment was performed according to the same procedure but without any stimulation.

### 2.5. Sample size

The purpose of the study was to compare the AS group and the NS group (control) through SEP measurement, which is the reaction index of EEG after electrical stimulation of the thumb and little finger, to check changes in the sensory threshold of the radial nerve and the ulna nerve by AS at LI4. However, no previous data were available that could be used to officially perform the test power calculation to determine the necessary sample size. Therefore, this study was designed as a pilot study to provide the initial data required to calculate the sample size for a large-scale clinical trial. By considering a 10% dropout rate in each group, 40 subjects (20 men, 20 women) were selected as the minimum sample size to provide statistical significance in a univariate analysis.

### 2.6. Analysis and outcome

**Figure 3:**
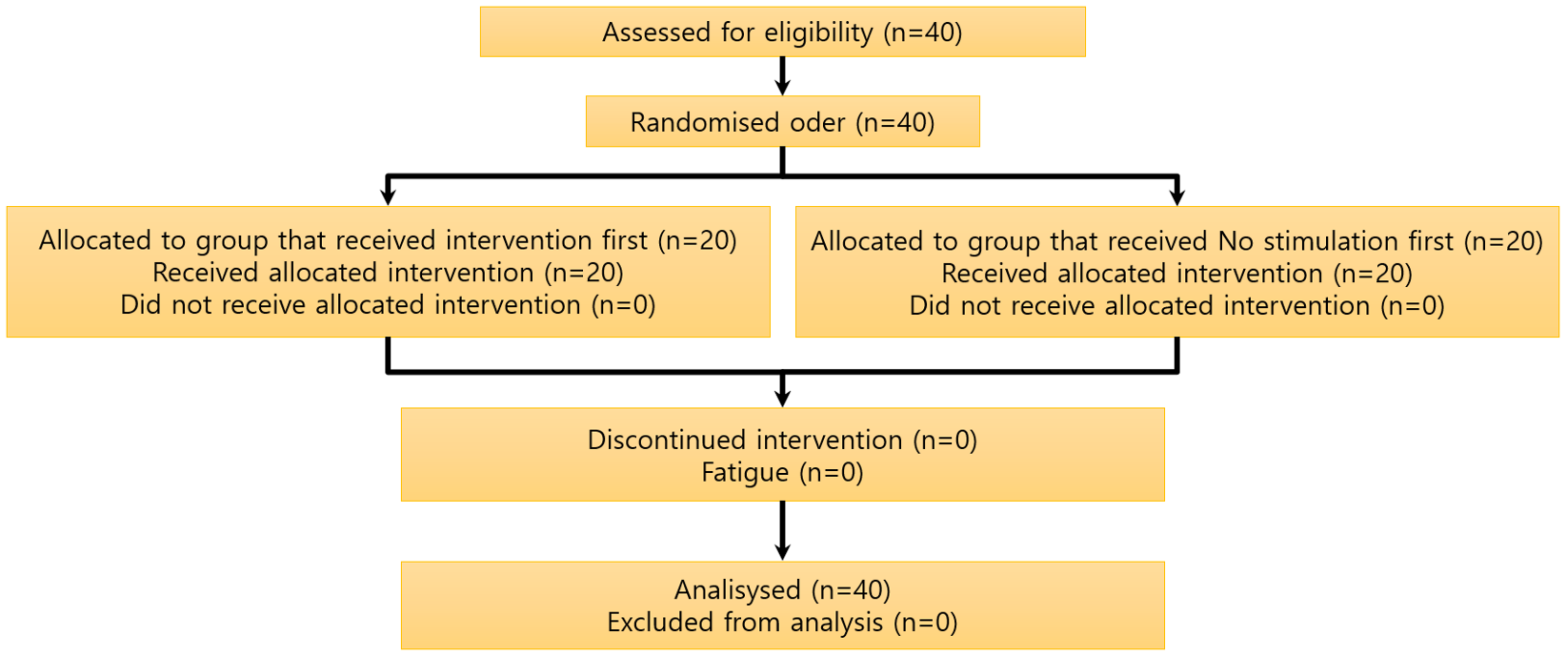
Participant flow chart. A total of 40 subjects were recruited, and there were no dropouts among the 40 subjects measured. The study was performed by dividing the 40 subjects into a control group and an acupuncture group, each group composed of 20 subjects, using the crossover method.

Of the 40 participants who were recruited, none dropped out, and all data obtained from them were analyzed as described below (Figure 3).

#### 2.6.1. SEP analysis (primary outcome)

An open-source license Python 3.8 (Python Software Foundation, United States) program (Van Rossum and Drake, 2009) was used for the SEP analysis, while an MNE-Python package was used for pretreatment and as the analytical tool (Gramfort et al., 2013). A bandpass filter was applied to the recorded SEP data to eliminate noise, and at this time, the lower and upper cutoff frequencies were 2 Hz and 50 Hz, respectively. Additionally, an independent component analysis was conducted to eliminate any artifacts. After separating the refined data into thumb and little finger stimulation data, 100 epochs with lengths ranging from −50 ms to 300 ms, on the basis of the stimulation point, were randomly extracted from each data point to obtain the mean value, and then the Savitzky–Golay filter was applied to smooth out the epochs.

**Figure 4:**
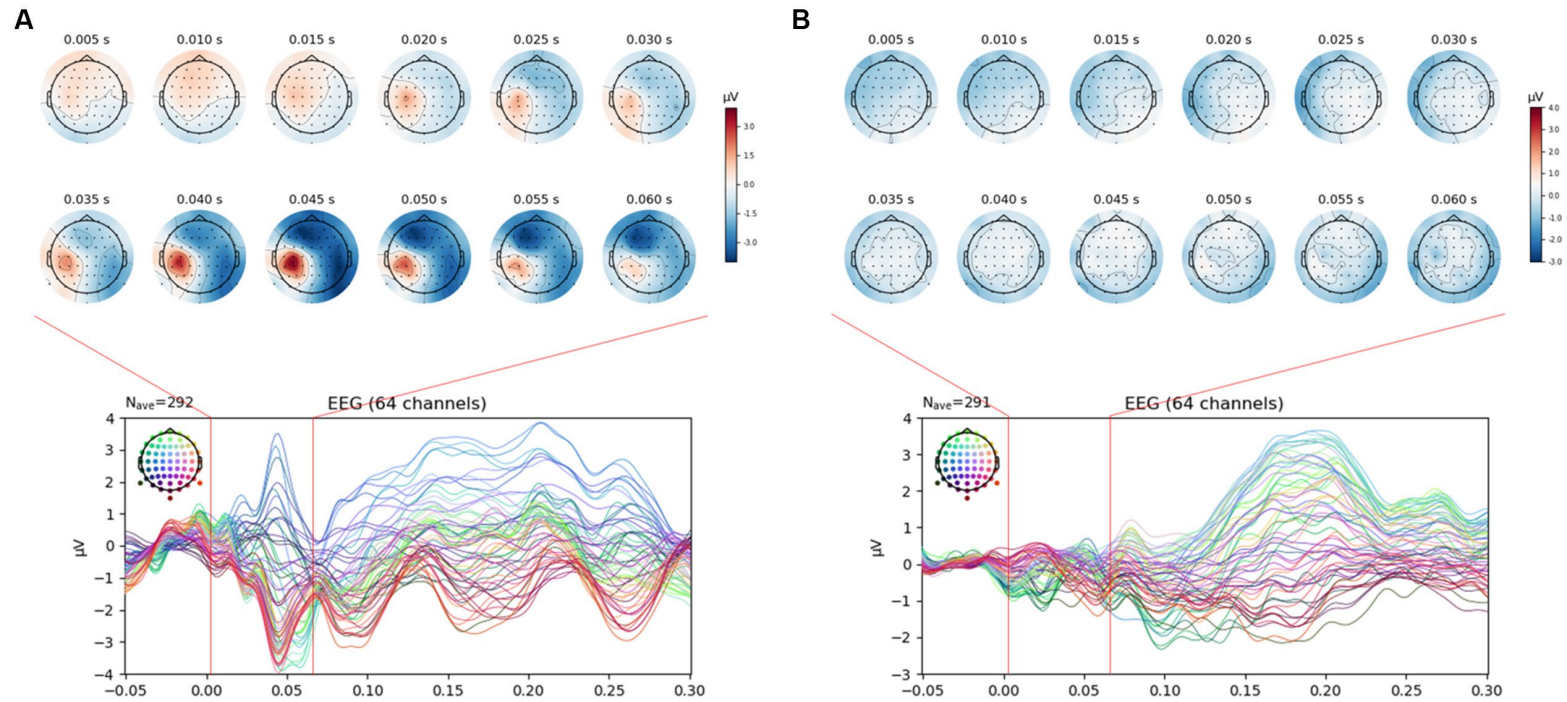
SEP manifestation after VRS-4 electrical stimulation of the thumb and little finger. **(A)**. Normal SEP manifestation; **(B)**. No SEP manifestation. In the study, only data with a normal SEP manifestation, as shown in A, were selected and analyzed for the EEG epoch analysis.

This experiment aimed to identify the effect of AS on the brain’s reaction to electrical stimulation applied to the thumb and little finger, and the recognition of electrical stimulation before intervention was a prerequisite. Figure 4 shows the SEP results measured before intervention in the control group, which was composed of different subjects. Electrical stimulation was recognized in the left parietal lobe area in (A) but not in (B). Thus, it was not possible to analyze data with no recognition of electrical stimulation before intervention, so they were excluded from the SEP analysis.

To compare the brain’s response to electrical stimulation of the thumb and little finger before and after intervention, a negative peak or positive peak was observed in the epochs after pretreatment at approximately 40 ms after electrical stimulation, and the peak difference (Δamplitude) was calculated for each subject by subtracting the peak value obtained before intervention from the peak value obtained after intervention. Then, the mean and the standard error for all subjects were calculated for each channel, and the peak difference was compared between “thumb vs. little finger” and “control vs. acupuncture.” Additionally, channels with a negative peak and channels with a positive peak were separated, and the mean and standard error for each peak channel were calculated to compare the overall pattern of peak changes. For the significance evaluation, the paired t test was conducted on the control and acupuncture groups using Δamplitude by each channel at the negative peak and positive peak of the thumb and little finger.

#### 2.6.2. Analysis of HRV and sensory threshold changes (secondary outcome)

For the HRV analysis, Low frequency (LF)/High frequency (HF) was calculated from the heart rate variability waveform using a Telescan program (Telescan, LAXTHA, KOREA), and the mean and standard error of LF/HF before and after intervention were calculated for all subjects. LF/HF is an indicator of the relative balance between sympathetic and parasympathetic nerves. Then, the LF/HF difference (ΔLF/HF) for each subject was calculated by subtracting the LF/HF value obtained before intervention from the LF/HF value obtained after intervention. The mean and standard error for all subjects were calculated, and the results obtained for the control and acupuncture groups were compared. To evaluate the significance, a paired t test was conducted on the control and acupuncture groups using ΔLF/HF.

For VRS, the mean and standard error of each electrical stimulation intensity value obtained before and after intervention were calculated for all subjects. Then, the intensity difference (Δintensity) for each subject was calculated by subtracting the intensity value before intervention from the intensity value after intervention, the mean and standard error for all subjects were calculated, and the VRS changes were compared between the thumb vs. little finger and the control vs. acupuncture group. For the significance evaluation, the paired t test was conducted on the control and acupuncture groups using Δintensity of the thumb and little finger.

## 3. Results

### 3.1. SEP amplitude

**Figure 5:**
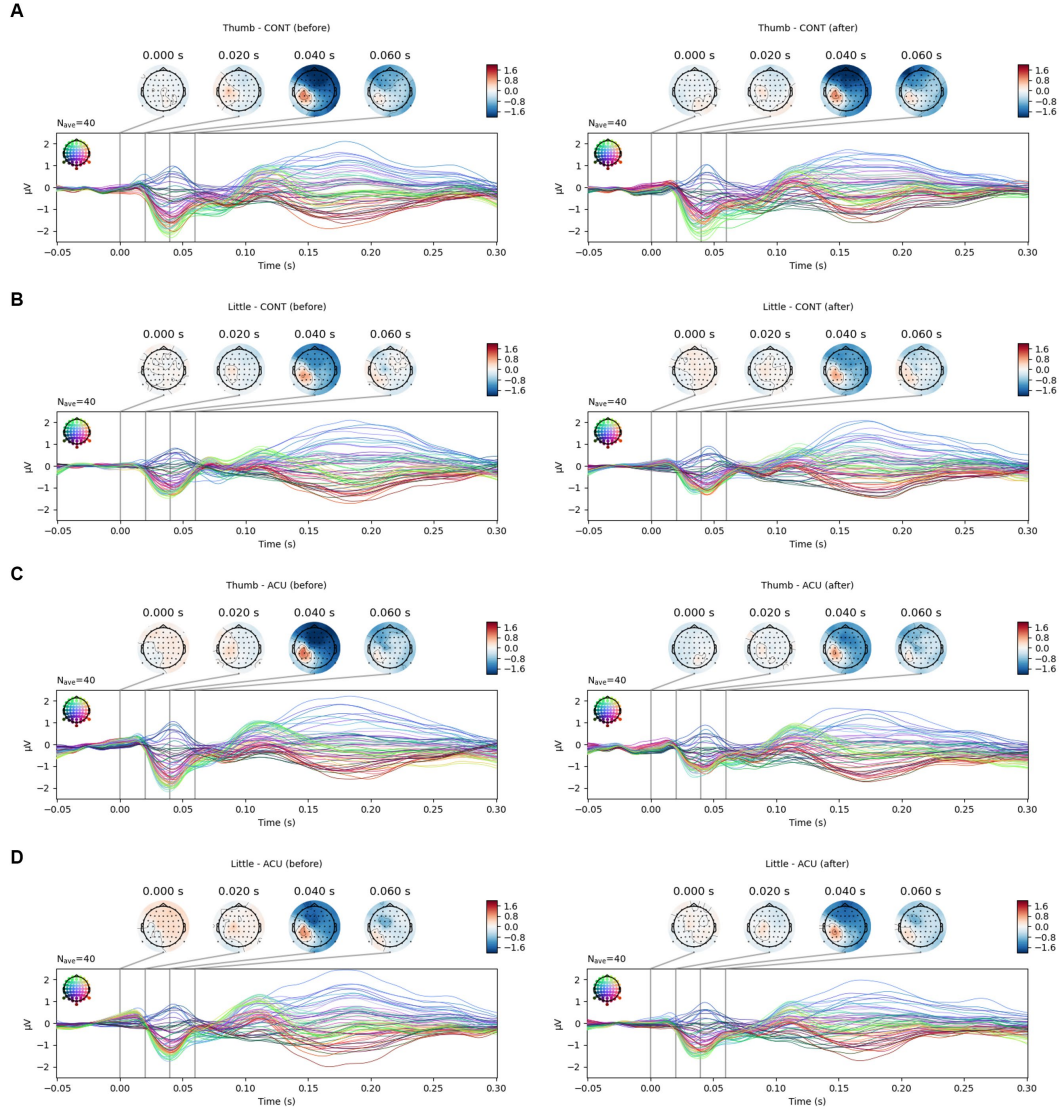
SEP changes in the thumb and little finger before and after intervention. **(A)**. SEP changes of the thumb in the control group; **(B)**. SEP changes of the little finger in the control group; **(C)**. SEP changes of the thumb in the acupuncture group; **(D)**. SEP changes of the little finger in the acupuncture group. It was confirmed that the SEP amplitude of the thumb (approximately 40 ms) decreased after AS.

**Figure 6:**
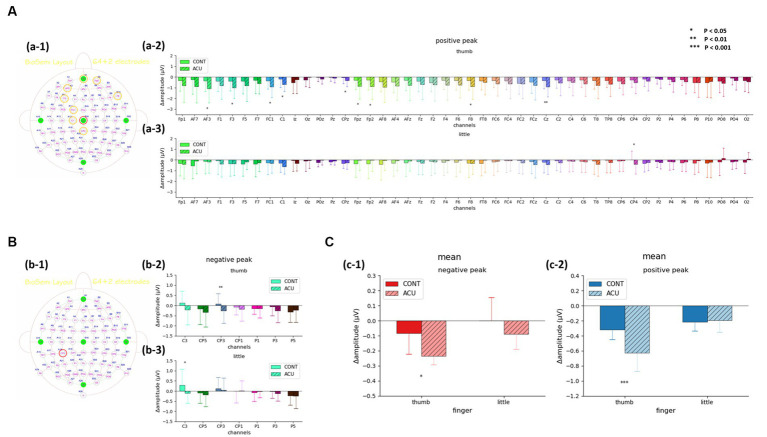
**(A)**. SEP amplitude changes of the positive peak before and after intervention at 40 ms in the acupuncture group and control group. (a-1) Channels with significant changes. (a-2) Thumb. (a-3) Little finger. **(B)**. SEP amplitude changes of the negative peak before and after intervention at 40 ms in the acupuncture group and control group. (b-1) Channels with significant changes. (b-2) Thumb. (b-3) Little finger. **(C)**. The mean value of SEP amplitude changes before and after intervention at 40 ms in the acupuncture group and control group. (c-1) Changes in the negative peak in the thumb and little finger. (c-2) Changes in the positive peak in the thumb and little finger. It was confirmed that changes in both the negative peak and the positive peak before and after intervention in the thumb decreased significantly in the acupuncture group compared to the control group.

For the SEP amplitude, it was confirmed that the size of the negative peak of approximately 0.04 s decreased after AS at Ll4 in the left parietal lobe area (Figure 5). When comparing SEP amplitude before and after intervention by the brainwave channels, at the positive peak, the SEP amplitude value for thumb stimulation decreased significantly in the AF3, F3, FC1, C1, CPz, Fpz, Fp2, F8 (all previous channels, *p* < 0.05) and Cz (*p* < 0.01) channels in the acupuncture group compared to the control group, and the SEP amplitude value for little finger stimulation decreased significantly at CP4 (p < 0.05) in the AS group compared to the control group. At the negative peak, the SEP value for thumb stimulation decreased significantly at the CP3 channel (*p* < 0.01) in the acupuncture group compared to the control group. The SEP for little finger stimulation decreased significantly at C3 (*p* < 0.05) in the AS group compared to the control group. When the all-channel mean of the SEP amplitude at the negative peak was calculated, it decreased significantly, with a *p* value at approximately 0.05 in the thumb only before and after intervention, while at the positive peak, the all-channel mean of the SEP amplitude decreased significantly, with a p value at approximately 0.001 in the thumb only (See Figure 6).

### 3.2. VRS and HRV

**Figure 7:**
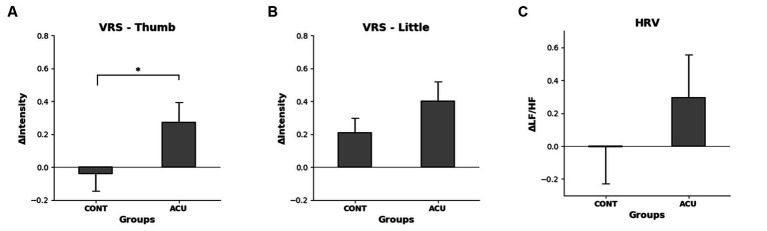
**(A)**. Changes in VRS 6 electrical stimulation intensity before and after intervention in the thumb of each group, **(B)**. Changes in VRS 6 electrical stimulation intensity before and after intervention in the little finger of each group, **(C)**. Difference in HF/LF values before and after intervention in each group. It was confirmed that the intensity of electrical stimulation increased significantly in the thumb but that HRV did not change significantly.

The VRS evaluation was conducted to determine the electrical stimulation intensity value for the degree of pain corresponding to VRS 6. While no difference was observed in the thumb before and after nonstimulation, the intensity of electrical stimulation was increased after AS, and the difference before and after intervention increased significantly in the AS group compared to the control group. In the case of the little finger, the electrical stimulation intensity value increased in both the control and AS groups after intervention, and the change was relatively high in the AS group, although the significance in both groups was not confirmed (See Figure 7).

The HRV measurement showed that the LF/HF value increased by 0.3 in the AS group compared to the control group, but not to a significant degree (See Figure 7).

## 4. Discussion

The somatic sensations of the human body are transmitted to the cerebrum via two ascending pathways; the dorsal column-medial lemniscal pathway and the spinothalamic pathway (Brooks and Tracey, 2005). The spinothalamic pathway is involved in pain sensation, and depending on the type of pain, it is transmitted along Aδ fibers (sharp, pricking pain, burning pain) or C fibers (freezing pain, burning pain) via the neo-spinothalamic tract and the paleo-spinothalamic tract, left–right crossing (decussation), and through the ventral posterior lateral nucleus to the cerebral cortex (Kevetter and Willis, 1984). These pain signals transmit information to four regions of the primary somatosensory cortex, including Brodmann’s areas 1, 2, 3a, and 3b, which are involved in sensory discrimination and intensity coding, process tactile object recognition at the secondary somatosensory cortex, and are involved in the perception, learning, and memory of pain-related events (Geyer et al., 2000; Schnitzler and Ploner, 2000). In this study, electrical stimulation corresponding to VAS 4, the level at which pain begins to be felt in the thumb and little finger of the right hand, was applied, and the response to pain observed around the left somatosensory cortex was experimentally confirmed through the SEP test. Desmedt et al. (1983) showed that the attention-related process can affect the early cortex SEP by identifying the evoked potential at approximately 40 ms as a response to target stimuli in an oddball task. The response to the target stimuli (electrical stimulation perceived by the subjects) was also observed as an evoked potential at approximately 40 ms, and data were randomly extracted for analysis, excluding all data without an SEP response for electrical stimulation of the fingers, to increase the reliability of the results of the SEP analysis of sensory stimulation responses.

For chronic pain, the analgesic effect of stimulation is better and its duration longer compared to acute pain because the analgesic effect is obtained through several sessions of stimulation of acupuncture points that are located at a relatively long distance from the pain area, or which are directly related; for acute pain, it is highly possible to manifest the analgesic effect locally at the stimulation site because it only takes a few seconds or minutes to obtain analgesic effects (Chang, 2013; Kelly and Willis, 2019). In the present study, pain was induced by applying electrical stimulation to the fingers to identify the analgesic effect of acupuncture for acute pain. When Ll4, which is included in the radial nerve, was stimulated, it was confirmed that the analgesic effect was manifested within a short period of time only in the thumb, which corresponds to the same radial nerve. This result suggests that AS can induce an analgesic effect at nearby nerves within a short period of time when acute pain develops.

In the study, MA was performed using the method of flicking the needle handle. MA generally activates A-type fibers and releases proinflammatory mediators such as PGE2,5-HT, histamine, bradykinin, and ATP to directly or indirectly stimulate nociceptors and activate C fibers (Zhao, 2008). Because AS directly stimulates two types of fibers in the radial nerve system, it was thought that the analgesic effect in the thumb, which is included in the radial nerve, would be manifested to a greater extent. The SEP amplitude for electrical stimulation of the thumb was decreased by AS within a short period of time, and in this case, it is thought that acupuncture was involved in the afferent nerve groups rather than the intracerebral mechanisms among the neurological mechanisms influenced by acupuncture. This study used manual stimulation to provide the most impactful acupuncture stimulation and conducted a study to compare before and after. Neurophysiological responses to the mere insertion of a needle were not considered. Therefore, it is necessary to conduct additional research to evaluate the neurophysiological response of acupuncture point stimulation response to needle insertion by dividing it into acupuncture point stimulation and sham stimulation.

The VAS or VRS is a tool used to evaluate the degree of pain subjectively. In this study, the intensity of electrical stimulation was checked by asking the participants to make a sound when they felt a subjective feeling corresponding to the VRS 6. As a result, it was confirmed that the intensity of electrical stimulation was increased significantly only in the thumb after AS at Ll4 compared to the nonstimulation group, which also supports the SEP results.

In studies on pain alleviation by acupuncture treatment, the role of the autonomic nervous system through HRV changes has been disputed. Lee et al. (2010) and Chung et al. (2014) concluded, from their systematic review of studies on humans, that there is no evidence for the HRV controlling effect of acupuncture and thus that additional studies would be needed, while the results of other studies contained evidence that acupuncture controls HRV (Anderson et al., 2012). In this study, HRV changes were checked by PPG measurements to evaluate the changes in the autonomic nervous system when the analgesic effect was attained through acupuncture treatment for acute pain. The tendency to activate sympathetic nerves could be identified by increased HRV values during acupuncture analgesia after inducing acute pain, but no significant changes were observed. From the above review articles, it can be surmised that it is not that AS does not affect HRV but that complex stimulation, along with the stimulation time and period and the intensity of the acupuncture point, can significantly affect HRV changes; thus, it is thought that the local AS for acute pain used in this study was not significantly effective with regard to HRV changes.

Psychological factors play an important role in pain control during acupuncture treatment (Thomas et al., 2016). However, this study was conducted without considering Psychological effects because it aimed to evaluate only the effect of acupuncture on the peripheral nerves for short-term, stimulation-induced acute pain, thus excluding psychological factors. Based on the results of the study, additional experiments are required in which psychological effect for acute pain are considered, as well as studies on the anatomically basic mechanism that supports the role of neurotransmitters in the ascending pathway and the descending pathway.

In conclusion, pain sensation is the interaction between sensory, psychological, and cognitive factors, and thus the study of the analgesic effect mechanism activated by acupuncture for the treatment of pain should be approached using more complex methods. Nevertheless, this study is meaningful in evaluating the range of the local nerve response induced by acupuncture treatment for acute pain. The results of this study suggest that acupuncture treatment for acute pain is effective when it directly stimulates the area where pain develops and induces an analgesic effect, because treatment is needed within a short period of time when pain develops, and there is a local response.

## Data availability statement

The original contributions presented in the study are included in the article/supplementary material, further inquiries can be directed to the corresponding author.

## Ethics statement

The studies involving humans were approved by Daejeon Korean Medicine Hospital affiliated with Daejeon University, Daejeon, Korea (IRB approval no. djomc-129-1). The studies were conducted in accordance with the local legislation and institutional requirements. The participants provided their written informed consent to participate in this study.

## Author contributions

K-HC: Conceptualization, Data curation, Formal analysis, Investigation, Methodology, Project administration, Software, Supervision, Validation, Visualization, Writing – original draft. SC: Data curation, Formal analysis, Software, Supervision, Validation, Visualization, Writing – review & editing. MK: Data curation, Formal analysis, Investigation, Methodology, Project administration, Writing – original draft. OK: Conceptualization, Data curation, Formal analysis, Methodology, Project administration, Writing – review & editing. S-YK: Formal analysis, Methodology, Writing – review & editing, Conceptualization. SS: Formal analysis, Writing – original draft. SB: Formal analysis, Writing – original draft. YR: Funding acquisition, Methodology, Project administration, Supervision, Writing – review & editing.

## Footnotes

^1^ https://cris.nih.go.kr/cris/search/detailSearch.do/6006
